# Detecting Partial Discharge in Cable Joints Based on Implanting Optical Fiber Using MZ–Sagnac Interferometry

**DOI:** 10.3390/s25103166

**Published:** 2025-05-17

**Authors:** Weikai Zhang, Yuxuan Song, Xiaowei Wu, Hong Liu, Haoyuan Tian, Zijie Tang, Shaopeng Xu, Weigen Chen

**Affiliations:** 1 State Key Laboratory of Power Transmission Equipment Technology, School of Electrical Engineering, Chongqing University, Chongqing 400044, China; wkzhang@stu.cqu.edu.cn (W.Z.); songyuxuan@cqu.edu.cn (Y.S.); liuhong@stu.cqu.edu.cn (H.L.); hytian@cqu.edu.cn (H.T.); tangzj0928@163.com (Z.T.); 2 Shandong Taikai Cable Co., Ltd., Tai’an 271000, China; taikaiwxw@163.com; 3 School of Mathematics and Statistics, Hainan University, Haikou 570228, China; 994284@hainanu.edu.cn

**Keywords:** optical fiber-implanted sensor, cable joint, partial discharges, MZ–Sagnac interferometry, acoustic detection

## Abstract

Detecting partial discharges in cable joints is critical for timely defect identification and reliable transmission system operation. To improve the long-term reliability and sensitivity of the sensing system, a novel method for cable joint monitoring based on implanting optical fibers within the joint structure is proposed. The electric field distribution of the optical fiber-implanted cable joint was simulated, followed by electrical performance tests, demonstrating that optical fiber implantation had a negligible effect on the electrical properties of the cable joint. A platform utilizing Mach–Zehnder–Sagnac (MZ–Sagnac) interferometry was developed to evaluate the frequency response of the implanted optical fiber sensor, with calibration performed on a non-standard curved surface. The results show that the average sensitivity of the sensor in the 10 kHz–80 kHz range is 71.6 dB, 2.0 dB higher than that of the piezoelectric transducer, with a maximum signal-to-noise ratio of 65.2 dB. To simulate common fault conditions in the actual operation of cable joints, four types of discharge defects were introduced. Partial discharge tests conducted on an optical fiber-implanted cable joint, supplemented by measurements using a partial discharge detector, demonstrate that the optical fiber sensors can detect a minimum discharge of 16.0 pC.

## 1. Introduction

Power cables are essential for transmitting electricity efficiently and reliably, supporting the stable operation of modern energy systems and the transition to renewable energy and smart grids [1,2,3]. Cable joints play a vital role by ensuring seamless connectivity and durability, maintaining uninterrupted power transmission across complex networks. However, as voltage levels increase and cables are in operation for extended periods, the insulation material at cable joints may develop local defects due to the combined effects of electrical and mechanical stresses. These defects often serve as the primary source of partial discharge (PD). In turn, sustained PD can exacerbate these defects, accelerating the degradation of the insulation material and, ultimately, leading to potential equipment failure [4,5,6]. Therefore, conducting PD detection for cable joints is significant for ensuring the safety of grid equipment and the reliable operation of the power grid, as it allows for the timely and effective identification of insulation defects at the cable joint locations [7].

The release of electrical currents, electromagnetic waves, acoustic signals, heat, light radiation, and various gases or chemical substances often accompanies PD in cables and their accessories [8]. As a result, PD detection methods can be broadly categorized into electrical and non-electrical techniques. Electrical methods, such as pulse current measurement and ultra-high-frequency detection, offer high sensitivity and real-time monitoring, but are prone to electromagnetic interference and may require complex installation [9,10]. Non-electrical methods, including ultrasonic testing [11,12] and temperature monitoring [13], provide non-invasive and adaptable solutions for PD detection in high-voltage environments. Temperature monitoring provides valuable insights into the type and severity of discharges but is less effective at detecting early-stage, small-scale discharges due to their susceptibility to environmental interference. Ultrasonic testing captures high-frequency sound waves generated by PD, providing reliable discharge detection in complex electrical environments due to its strong resistance to electromagnetic interference [14,15,16].

As a traditional ultrasonic sensor, the piezoelectric transducer (PZT) is characterized by its simple structure and is widely used for PD detection in gas-insulated switchgear and transformers [17,18,19]. However, due to their relatively large size, PZTs are challenging to deploy effectively in cable systems. Consequently, researchers have continuously sought ultrasonic detection methods that are easy to integrate and simple to install. In recent years, the rapid advancement of optical fiber sensor (OFS) technology has introduced optical fiber ultrasonic sensors as a promising alternative. These sensors, with their high sensitivity, immunity to electromagnetic interference, compact size, and ease of integration into power equipment, effectively address the limitations of traditional detection methods [20,21].

The weak acoustic waves generated by PD alter the parameters of light propagation within the OFS, coupling the acoustic wave information into the optical signal, which is then transmitted to the detection device. Consequently, the OFS enables the detection of PD [22,23]. In order to enhance the reliability of power equipment operation, an increasing number of researchers have focused on the application of optical fiber ultrasonic sensors in the detection of PD in cables and joints. Qin Weiqi et al. employed a COTDR system to detect and locate PDs in long-distance cables, utilizing two erbium-doped fiber amplifiers to achieve a signal-to-noise ratio (SNR) of 24.45 dB at a distance of 20 km [24]. Che Qian et al. proposed a φ-OTDR system that provides a detection method for PD in XLPE cables [25]. A weak fiber grating significantly improves the SNR, achieving an impressive 58 dB. Distributed optical fiber sensing technology offers the advantage of long sensing distances, making it particularly effective for monitoring cable systems. However, it often suffers from low sensitivity and limited spatial resolution. In contrast, quasi-distributed optical fiber sensing systems are better suited for cable joints characterized by short sensing distances and frequent partial discharges. Cheng Yanting et al. proposed a cable PD sensor based on BOTDR, which achieves multi-point PD detection by embedding the optical fiber between the cable’s water-blocking tape [26]. Sun Yuwei et al. employed a fiber loop configuration to develop an ultrasonic detection system for PD detection at cable joints [27]. In measuring joint misalignment discharges, the system was capable of detecting a minimum discharge level of 40 pC to 50 pC. Liu Zhiheng et al. designed an axial core ultrasonic sensor based on an optical fiber Sagnac interferometer, which is used for PD detection in high-voltage direct current cable systems [28]. The sensor operates at center frequencies of 25 kHz, 58 kHz, and 175 kHz, allowing real-time PD detection in cables without affecting their operational state. To address the noise amplification caused by birefringence, the MZ–Sagnac interferometer (MZ-SI) employed in this study incorporates a Faraday rotator mirror, which effectively eliminates birefringence and enhances the stability of the system.

As the core component of an optical fiber sensing system, the sensor plays a critical role in signal perception, conversion, and transmission. Due to the short distance and frequent discharges at cable joints, a higher sensitivity and SNR are required compared to cable structures. However, most existing sensors are primarily based on external wrapping or axial core designs, with the sensing head positioned outside the cable joint, far from the partial discharge source. This limitation prevents the detection of small discharge signals at the early stages of faults. Additionally, challenges in on-site installation hinder the full utilization of the inherent advantages of OFS, such as compact size, corrosion resistance, and complete electrical insulation. Due to the unique structure of the cable joint, it facilitates the integration of the optical fiber sensor. Implanting the optical fiber into the cable joint brings it closer to the discharge source, thereby enhancing sensitivity [29,30]. Additionally, after implantation, the cable joint can shield part of the external noise, improving the SNR.

Therefore, this paper proposes an optical fiber-implanted cable joint based on the MZ-SI, which integrates sensing and insulating functions to detect PD at the cable joint. In addition, finite element simulation analysis is conducted to examine the impact of optical fiber implantation on the electric field distribution at the cable joint. The power frequency withstand voltage test and PD test are designed to ensure the insulation performance of the optical fiber-implanted cable joint. The internal acoustic field response of the cable joint is experimentally measured using an optical fiber-implanted sensor and compared with the sensitivity of the PZT. Furthermore, a real cable joint PD experimental platform is constructed, enabling ultrasonic signal detection for point discharge, air gap discharge, discharge along the surface, and joint misalignment through the optical fiber-implanted cable joint.

## 2. Optical Fiber MZ-SI Ultrasonic Sensing System

### 2.1. Principle of Optical Fiber MZ-SI Sensing

Optical fiber interferometric ultrasonic sensing technology utilizes phases for sensing with a high sensitivity, fast response time, simple system, and low cost. The optical path structures can be divided into four basic types: a Michelson interferometer, a Mach–Zehnder interferometer, a Sagnac interferometer, and a Fabry–Perot interferometer [31]. Although fiber optic interferometric sensors based on a single optical path structure offer many advantages, they all face challenges that are difficult to resolve [32]. In 1996, Fang proposed MZ-SI for the first time by combining a Mach–Zehnder interferometer and a Sagnac interferometer [33]. This method is widely used in PD detection due to its advantages of good stability, multiplexing, anti-interference ability, and high-frequency detection capability.

**Figure 1:**
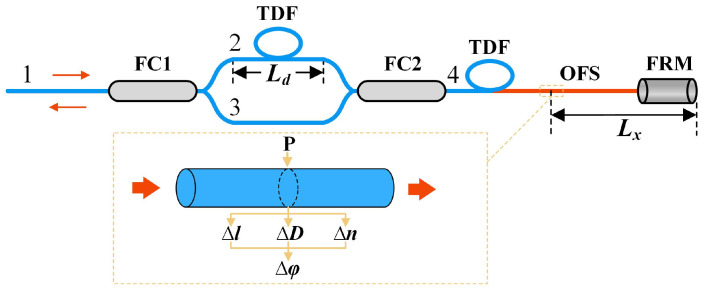
Basic structure of optical fiber MZ-SI (FC1, FC2: fiber coupler; TDF: time-delay fiber; OFS: optical fiber sensor; FRM: Faraday rotator mirror).

The basic structure of the fiber-based MZ-SI is illustrated in Figure 1. The light emitted by the laser is divided into two beams through coupler FC1. The first beam passes through the TDF and reaches coupler FC2, while the second beam directly reaches FC2, where both beams merge into a single beam. This combined beam then passes through the optical fiber and is reflected by the FRM. Afterward, in FC2, the light is split into two separate beams again, which, respectively, pass through the TDF and reach FC1. Finally, the light interferes at FC1 and is sent out through the input. There are a total of four possible light propagation paths, which can be described as follows:

(a) 1-2-4-FRM-4-2-1;
(b) 1-2-4-FRM-4-3-1;
(c) 1-3-4-FRM-4-2-1;
(d) 1-3-4-FRM-4-3-1.

According to the principle of optical fiber interference, interference occurs when the optical path lengths of paths 1 and 2 are equal. If the optical path length differences between the other two paths and either path 1 or path 2 significantly exceed the coherence length of the light source, no interference will take place, and only part of the DC component of the signal will be formed. The coherence length of the light source can be calculated using the following equation:

(1) $$L_{C}=\frac{\lambda^{2}}{\Delta \lambda }$$

where λ denotes the center frequency of the light source and Δλ represents its full width at half maximum.

An optical fiber with length l, refractive index n, and wavelength λ transmits light through the fiber. The phase φ of the light can be expressed as

(2) $$\varphi =\frac{2\pi nl}{\lambda }=\beta l$$

where β is the propagation constant. According to the photoelastic effect, strain effect, and Poisson effect, when the ultrasonic signal reaches the OFS, it will be in the form of acoustic pressure to change the core refractive index of the optical fiber n, the fiber length l, and the fiber diameter D, resulting in the light in the optical fiber in the amount of the phase change being

(3) $$\Delta \varphi =l\left(\frac{\partial \beta }{\partial n}\right)\Delta n+\beta l\frac{\Delta l}{l}+l\left(\frac{\partial \beta }{\partial D}\right)\Delta D$$

where the optical fiber diameter D is affected slightly, ignoring the Poisson effect, and when combined with the generalized Hooke law, it can be obtained as follows:

(4) $$\Delta \varphi =\beta lP_{s}(1-2\mu )\left[\frac{n^{2}(p_{l}+p_{t})}{2}-1\right]/E$$

where $P_{s}$ is the acoustic pressure of ultrasound reaching the sensing fiber, μ is the Poisson ratio of the optical fiber, $p_{l}$ and $p_{t}$ are the strain photoelastic coefficients, and E is the elastic modulus of the fiber. According to Equation (4), Δφ is proportional to $P_{s}$. Therefore, the magnitude of the acoustic pressure can be obtained by demodulating the phase information. As shown in Figure 1, when the external acoustic pressure P is applied at position $L_{x}$, the phase difference between path 1 and path 2 is

(5) $$\Delta \phi (t)=\phi_{1}(t)-\phi_{2}(t)=\left[\Delta \varphi (t)+\Delta \varphi (t-2\tau_{x})\right]-\left[\Delta \varphi (t-\tau_{d})+\Delta \varphi (t-\tau_{d}-2\tau_{x})\right]$$

where $\tau_{x}$ and $\tau_{d}$ represent the propagation time of light in $L_{x}$ and $L_{d}$, respectively.

### 2.2. Dual-Path Optical Fiber MZ-SI System Construction

**Figure 2:**
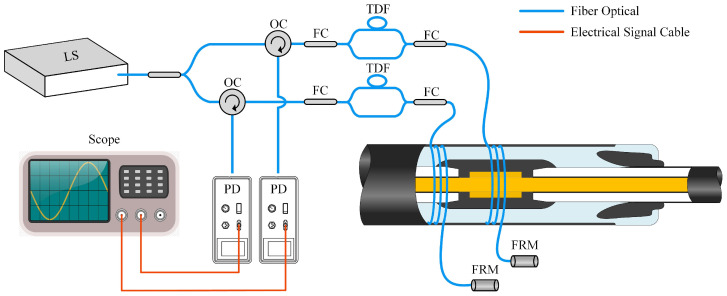
Optical fiber MZ-SI cable joint sensing system.

The sensing system based on the dual-path optical fiber MZ-SI, as shown in Figure 2, has been constructed in this research, integrating the sensing optical fiber with a cable connector. The optical fiber is implanted inside the outer shield of the cable connector, and the detailed process of preparing the optical fiber-implanted cable joint is described in Section 3.2 of this paper. In a fiber optic system, source instability can lead to variations in interference intensity, increased noise, and consequently, a reduction in the SNR. Moreover, long-term system stability may degrade, adversely affecting measurement accuracy. To enhance source stability, the system utilizes a 20 mW low-noise, ultra-narrow linewidth 1550 nm laser as the light source. Additionally, the system design has been optimized by mitigating regions with excessively small fiber bending radii, thereby reducing signal loss and further improving overall stability.

A 3 dB coupler splits the light into two beams, which serve as light sources for sensors located at different positions. The light source enters through port 1 of the circulator, travels to the 3 dB coupler via port 2, and is divided into two beams. The circulator’s structural design prevents damage to the laser from reflected light. Two identical sensing circuits are constructed to simultaneously detect PD signals at different locations of the cable connector. Faraday mirrors are connected to the ends of both sensing circuits to ensure that the polarization direction of the incident light is rotated by 90°. The light from path 1 and path 2 interferes at the 3 dB coupler, and the interference intensity can be expressed as follows:

(6) $$I(t)=2|E_{0}{|}^{2}\left[1+\cos(\Delta \phi (t)+\phi_{0})\right]$$

where $E_{0}$ is the optical field amplitude and $\varphi_{0}$ is the system’s intrinsic phase difference. The interfering light enters the photodetector through the circulator port 3, and the optical signal is converted into an electrical signal. Since the cable joint PD ultrasonic distribution is below 100 kHz, the electrical signal must be filtered, amplified, and then displayed and stored by an oscilloscope.

## 3. Performance Testing of an Optical Fiber-Implanted Cable Joint

### 3.1. Electric Field Distribution of an Optical Fiber-Implanted Cable Joint

**Figure 3:**
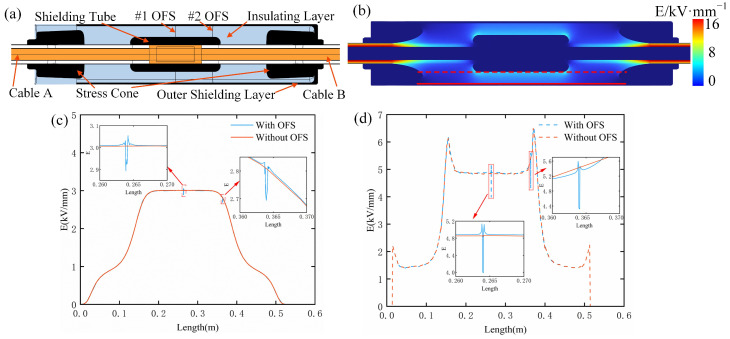
Cable joint model diagram and internal electric field distribution: (**a**) three-dimensional model of the optical fiber-implanted cable joint; (**b**) overall electric field distribution diagram; (**c**) electric field distribution curve along the optical fiber implantation position between the insulation layer and the outer shielding layer; (**d**) electric field distribution curve along the optical fiber implantation position between the insulation layer and the shielding tube.

The structure of the 110 kV prefabricated cable joint, as shown in Figure 3a, is composed of a high-voltage shielding tube, two stress cones, an insulating layer, and an outer shielding layer. The insulating layer is made of insulating silicone rubber, while the high-voltage shielding tube, stress cones, and outer shielding layer are fabricated from semi-conducting silicone rubber. Cable joints connect cable A to cable B and have the advantage of high electrical reliability and easy installation. The high-voltage shielding tube serves to shield the electric field, preventing its concentration. The insulating layer is a critical electrical insulation. The stress cones improve the electric structure that ensures the cable’s operation by providing field distribution at the cable’s termination and reducing the electric field intensity at the edges. The outer shielding layer protects against external electromagnetic interference and shields the internal components from mechanical damage and environmental factors. The manufacturing process of the cable joints is as follows: the high-voltage shielding tube and stress cones are vulcanized and molded, followed by an injection molding process to form the insulating layer, and finally, the outer shielding layer is formed through the final injection molding.

Based on the above analysis, it is concluded that the outer shielding layer has the minimal impact on the distribution of the electric field within the shield. To ensure the electrical performance of the cable joint, this study proposes a method for implanting the optical fiber between the insulating layer and the outer shielding layer of the cable joint. This paper performs a finite element simulation of the cable joint to further specify the effect of optical fiber-implanted sensors on the electric field distribution inside the cable joint and establishes a three-dimensional model of the optical fiber-implanted cable joint location. As shown in Figure 3a, the optical fiber-implanted cable joint at the middle position is designated as the #1 OFS, while the one implanted at the end position is designated as the #2 OFS. Under alternating voltage, the electric field distribution at the cable joints is primarily determined by the relative permittivity and electrical conductivity of the materials. The governing equation used in this study is as follows:

(7) $$\nabla \cdot D=\rho_{V}$$

(8) $$D=\varepsilon_{0}\varepsilon_{r}E$$

(9) E=−∇V

where D is the electric displacement vector, $\rho_{V}$ is the local charge density, $\varepsilon_{0}$ is the permittivity of the vacuum, $\varepsilon_{r}$ is the relative permittivity of materials, E is the electric field vector, and V is the potential. The dielectric parameters of the materials at the cable joints are shown in Table 1 [9,34].

The simulation primarily investigates the distribution of the electric field inside the cable joint under the steady-state industrial frequency (50 Hz) condition, with both the cable’s outer shield and the cable joint’s outer shield grounded, and a voltage of 110 kV applied to the conductor. The overall electric field distribution of the cable joint is illustrated in Figure 3b. The insulation layer of the cable joint mitigates electric field concentration and exhibits a characteristic gradient, with the electric field strength gradually decreasing from the center to the outer regions. Since the cable insulation plays a major role in alleviating electric field concentration, higher electric field strengths are observed at areas of structural distortion, such as near the stress cone and the high-voltage shielding tube. The maximum electric field strength near the stress cone is 8.92 kV/mm, while near the high-voltage shielding tube, it reaches 6.41 kV/mm.

Since each structural component of the cable joint is formed through a single casting process, the optical fiber can only be implanted between two adjacent structures, namely, between the insulation layer and the outer shielding layer and between the insulation layer and the shielding tube. As shown by the red line in Figure 3b, the electric field distributions before and after the implantation of optical fibers were compared along these two positions. As shown in Figure 3c, implanting the optical fiber between the insulation layer and the outer shielding layer slightly increases the electric field strength within the cable joint. Specifically, implanting the #1 OFS caused a slight distortion in the electric field, with the field strength increasing from 3.01 kV/mm to 3.06 kV/mm. Implanting the #2 OFS led to an increase in the electric field value from 2.81 kV/mm to 2.83 kV/mm. As shown in Figure 3d, implanting the optical fiber between the insulation layer and the shielding tube results in a more pronounced increase in electric field strength and causes more significant electric field distortion. Specifically, implanting the #1 OFS increased the electric field from 4.86 kV/mm to 5.13 kV/mm, while implanting the #2 OFS raised the field strength from 5.38 kV/mm to 5.59 kV/mm. These results indicate that implanting the optical fiber between the insulation layer and the outer shielding layer has a negligible effect on the internal electric field distribution of the cable joint.

### 3.2. Preparation and Electrical Performance Testing of the Optical Fiber-Implanted Cable Joint

**Figure 4:**
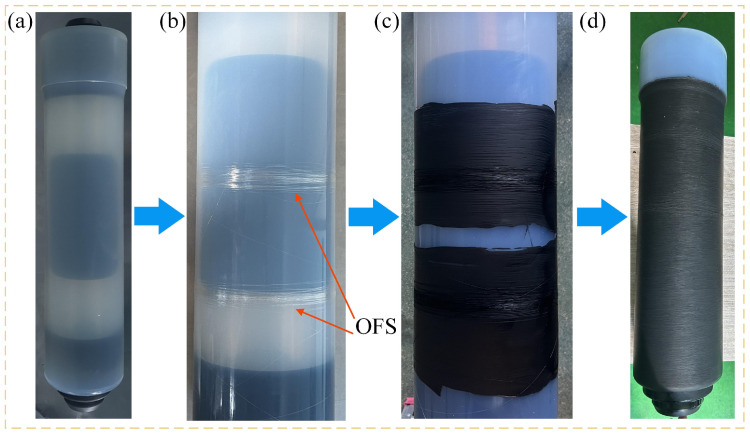
Preparation process of the optical fiber-implanted cable joint: (**a**–**c**) preparation process of the optical fiber-implanted cable joint; (**d**) finished product image of the optical fiber-implanted cable joint.

Based on the method of implanting optical fibers between the insulating layer and the outer shielding layer of the cable joint, this study fabricated an optical fiber-implanted cable joint. The detailed preparation process is illustrated in Figure 4. First, a semi-finished product without the outer shield is prepared. The optical fiber is then wound around the outer surface of the cable’s insulation layer. The fiber end is looped around the joint insulator and extracted through the end of the cable joint, as shown in Figure S1. This extraction method increases the bending radius of the fiber, enhancing the structural stability of the optical fiber. Semi-conductive silicone rubber is vulcanized at the locations where the optical fiber is wound, followed by a secondary vulcanization of semi-conductive silicone rubber over the entire outer surface of the insulation layer. Figure 4d presents a physical depiction of the optical fiber-implanted cable joint. In the interferometric OFS, the length of the sensing fiber is critical: a fiber that is too short results in reduced detection sensitivity, while an excessively long fiber can deteriorate the SNR [35]. In this study, two rolls of optical fiber, each with 80 turns, are wound at different positions: one at the center and the other 10 cm away from the center. The objective is to investigate the propagation characteristics of ultrasonic signals at these two positions.

**Figure 5:**
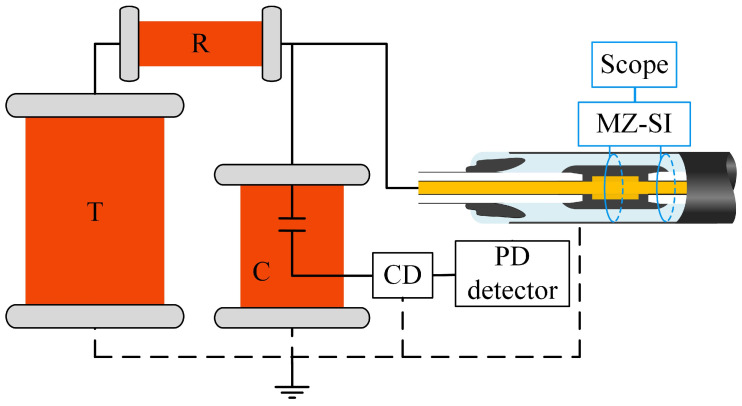
True-type cable joint test platform (T, test transformer; R, protection resistance; C, capacitive voltage divider; CD, coupling device).

To investigate the effect of optical fiber implanted inside the cable joint on its insulation, the power frequency withstand voltage test and PD test are carried out. The true-type cable joint test platform is shown in Figure 5, and the experimental platform includes a no-local-discharge pressurization platform, a PD detection circuit, and a cable joint test platform. The cable conductor is connected to the power supply, and the cable’s outer shield and the cable joint’s outer shield are grounded. The power supply is boosted to 160 kV (2.5U0) and kept for 30 min, and no breakdown occurs in the cable joint; the voltage is reduced to 96 kV (1.5U0), and the amount of PD is 4.1pC, which is less than the 5pC required by the standard. The result is shown in Figure S2. Then, the cable joint passes the test of the standard [36].

Although implanting optical fibers into cable joints still faces challenges, such as difficulties in automation, high process requirements, and high maintenance demands, the results from the electric field simulation, power frequency withstand voltage tests, and partial discharge tests lead to the conclusion that optical fiber implantation has a negligible impact on the electric field distribution and insulation performance of the cable joint. This indicates that despite certain technical challenges, optical fiber implantation can effectively monitor while maintaining the stability of the electrical performance.

### 3.3. Response Characteristics of Optical Fiber Ultrasonic Sensors

**Figure 6:**
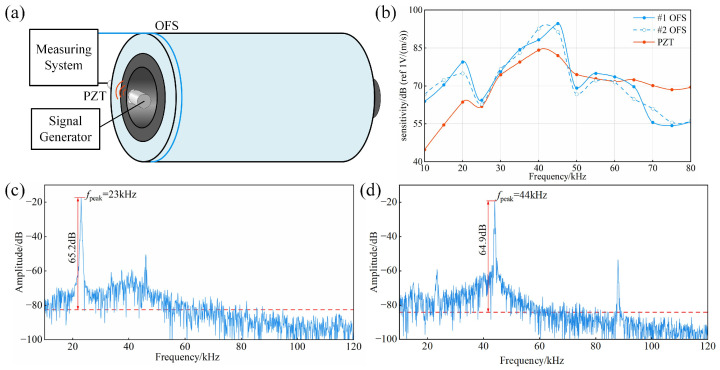
Schematic diagram and the experimental results of acoustic field response characteristics inside the cable joint: (**a**) structural schematic diagram; (**b**) frequency response curves of the OFS and PZT; (**c**) the SNR of the OFS at 23 kHz; (**d**) the SNR of the OFS at 44 kHz.

A platform for characterizing the internal sound field response of the cable joint is constructed, as shown in Figure 6a, and the optical fiber MZ-SI ultrasonic sensing system, as shown in Figure 2, is used to probe the frequency response of the internal sound field of the cable joint in comparison with the commercial PZT. The test platform includes a signal generator, an acoustic emission transducer (FUJI, REF-VL), an optical fiber-implanted cable joint, and a PZT (PENGXIANG, PXR04). The signal generator sends sinusoidal signals of different frequencies to the acoustic emission sensor, which generates sinusoidal acoustic signals of the same frequency. Both the OFS and PZT were placed outside the insulating layer of the cable joint to capture the acoustic signal inside the cable joint, and the signals were stored using an oscilloscope.

Referring to the longitudinal frequency response calibration method in the “JJF 1337-2012 Calibration Specification for Acoustic Emission Sensors (Comparative Method)” [37], the signal generator should produce a pulse with a width less than 1 μs and an amplitude greater than 5 V, and when the reference and test sensors are placed at the same position, they are assumed to receive identical acoustic signals. An acoustic emission sensor was placed inside the cable joint, and an acoustic emission couplant was applied to its surface to simulate the ultrasonic signal generated by the discharge. Unlike the standard procedure described in [34], and in order to accommodate the structural constraints of the cable joint and better reflect practical engineering applications, the PZT was positioned on the outer surface of the insulating layer of the cable joint. An acoustic emission couplant was applied to the PZT surface to ensure effective acoustic coupling. Although the contact surface was non-standard, the PZT and the OFS were placed at the same location. Therefore, it can be reasonably assumed that both sensors received the same acoustic signal, which satisfies the fundamental requirement of the comparative calibration method. Under the same experimental conditions, experiments were conducted separately on the #1 OFS and #2 OFS.

Based on the calibration method given in [37], the sensitivity $S_{1}\left(f_{m}\right)$ of the commercial PZT at a signal frequency of $f_{m}$ is known; then, the sensitivity of the OFS is

(10) $$S_{2}(f_{m})=\frac{U_{2}(f_{m})}{U_{1}(f_{m})}S_{1}(f_{m})$$

where $U_{1}\left(f_{m}\right)$ represents the frequency response of the PZT at frequency $f_{m}$, while $U_{2}\left(f_{m}\right)$ represents the frequency response of the OFS at frequency $f_{m}$.

After the experiment, the frequency responses of the sensors in the 10 kHz~80 kHz range were obtained, with the frequency response curves shown in Figure 6b. The #1 OFS is most sensitive at 45 kHz, with a sensitivity reaching 94.6 dB. Its sensitivity generally exceeds that of the PZT in the low-frequency range of 10 kHz~60 kHz. The average sensitivity of the #1 OFS over the 10 kHz~80 kHz test band is 71.6 dB, which is 2.0 dB higher than that of the PZT, and the average sensitivity of the #2 OFS is 71.2 dB. The frequency response curves indicate that the two OFSs exhibit good consistency. The sensor’s frequency band is defined by a 3 dB amplitude attenuation, with the #1 OFS operating in the range of approximately 12 kHz to 66 kHz and the #2 OFS operating in the range of approximately 10 kHz to 64 kHz. Since the ultrasonic frequency of the PD lies in the 0~50 kHz range [24,28], the signal generator was adjusted so that the acoustic emission sensor emitted 23 kHz and 44 kHz signals. The SNR of the OFS was then measured, as shown in Figure 6c,d. In calculating the SNR, the noise level was defined as the average power computed over the entire frequency spectrum, which is represented by the red line in the figure, ensuring a comprehensive representation of the background noise. For the 23 kHz ultrasonic signal, the peak frequency of the #1 OFS was 23 kHz, with an SNR of 65.2 dB. For the 44 kHz ultrasonic signal, the peak frequency of the #1 OFS was 44 kHz, with an SNR of 64.9 dB. The response of the OFS is consistent with the ultrasonic signals, indicating that the optical fiber-implanted cable joint has a better SNR and higher accuracy. This is beneficial for the measurement of PD signals using an optical fiber-implanted cable joint, establishing a strong experimental foundation for such measurements.

## 4. PD Detection Test and Analysis Based on an Optical Fiber-Implanted Cable Joint

### 4.1. True-Type Cable Joint PD Test Platform

In this study, a true-type cable joint test platform, as illustrated in Figure 5, is utilized to evaluate the effectiveness of the OFS in detecting PD signals within the cable joint. During actual operation, improper cleaning of copper shavings during installation can lead to point discharges and discharges along the surface of the cable joint. Damage to the cable body when handling it during installation leads to air gap discharges. Cable joints run for a long time, resulting in the loss of elasticity of the silicone rubber material and insufficient interference, resulting in discharge along the surface. Additionally, improper installation can cause joint misalignment, triggering discharges [5,9,38]. To simulate PD defects in cable joints, four defect models are established: point discharge, air gap discharge, discharge along the surface, and joint misalignment discharge.

**Figure 7:**
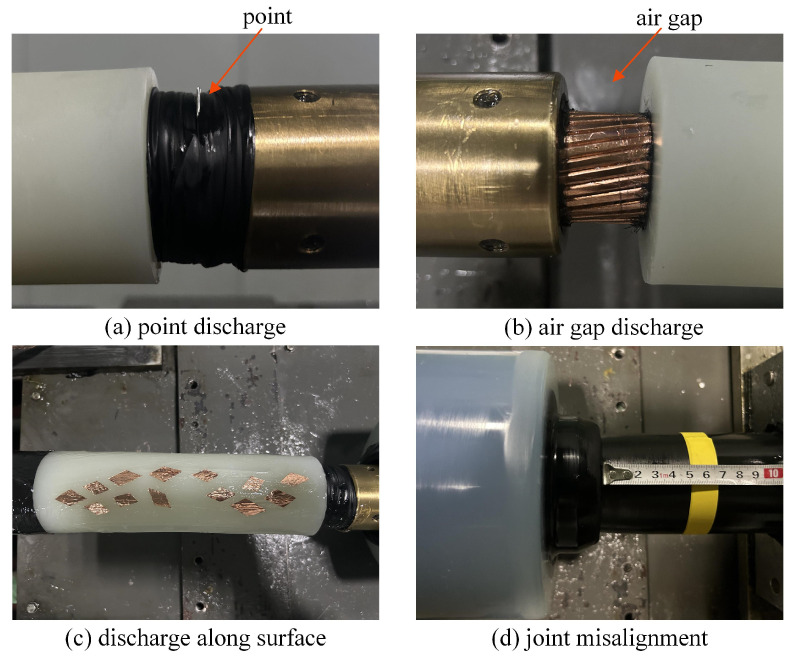
Actual images of four types of defects.

A metal wire is inserted into the semi-conducting strip near the conductor of cable A as a point defect, and the metal wire length is set to 1 cm, as shown in Figure 7a. Then, remove the semi-conducting tape on one side of the conductor of cable A to form an annular air gap defect with a length of 2 cm and a depth of 0.5 cm, as shown in Figure 7b. Netx, paste several rhombic copper sheets with an area of about 1 cm^2^ on the surface of the outer insulation layer of the cable A, which is intended to induce the phenomenon of discharging along surface between the conductor and the outer shield of the cable in the process of pressurization, as shown in Figure 7c. Finally, move the position of the cable joint 5 cm towards the direction of cable B to form a joint misalignment defect, as shown in Figure 7d. The initial discharge amounts for these defects were measured using a PD detector, with values of 16.7 pC, 12.4 pC, 11.7 pC, and 20.0 pC, respectively.

According to standards [39,40], a preliminary calibration test was carried out on the PD detector before the experiment commenced. A calibrator was used to inject a standard calibration charge of 50 pC into the cable sample, and the PD detector was adjusted to display a corresponding measurement of 50 pC. After the calibration was completed, the calibrator was removed from the circuit. The PD experiment was performed using a stepwise voltage increment method, with an initial voltage step of 2 kV. At each voltage level, the applied voltage was maintained for 20 s. Once the PD detector indicated a discharge magnitude exceeding 5 pC, the voltage step was reduced to 1 kV, and the holding time was increased to 1 min. When a discharge signal was observed in the OFS system, the voltage was no longer increased. The signals detected by the OFS system were then compared with those recorded by the PD detector to ensure that the detected signals were not artifacts or noise. If the OFS system was confirmed to have detected the PD signals, the total PD magnitude recorded by the OFS system within 1 min was averaged. This averaged value was defined as the smallest average discharge amount detectable by the OFS within one minute.

To explore the relationship between the sensitivity of the OFS in detecting PD and axial distance, and to ensure that the PD signal source is from a defect, this paper records signal data from two OFSs. In Figure 3a, the signal generated by sensor #1 is labeled as the #1 signal, and the signal generated by sensor #2 is labeled as the #2 signal, with the defect located closer to the #1 OFS. Due to the need for ultrasonic coupling agent application at the location of the piezoelectric ceramic in the PZT during practical installation, and its lower sensitivity in the low-frequency range compared to the OFS, only the OFS is used for detecting partial discharge in the cable joint.

### 4.2. PD Results of Point Defect Tests

**Figure 8:**
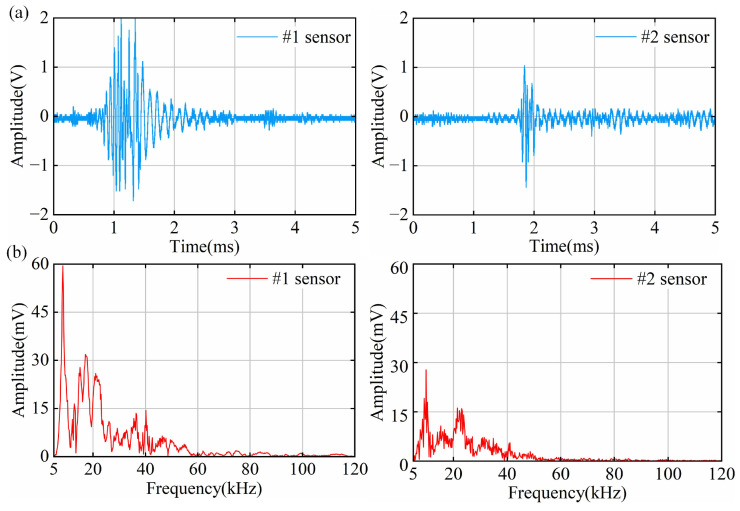
Time–frequency domain signals of PD in point defects: (**a**) time–domain signal diagram; (**b**) frequency–domain signal diagram.

The smallest average discharge amount detectable by the OFS within one minute is 48.4 pC. The time–domain waveforms of the point discharge signals measured by the OFS are shown in Figure 8a. The amplitude of the #1 signal is greater than that of the #2 signal, and the #1 signal also exhibits a longer duration. This behavior can be attributed to the fact that the main material of the cable joint is silicone rubber, which has a significantly higher acoustic attenuation coefficient compared to metal [41]. The attenuation of partial discharge signals within the cable joint is substantial and cannot be neglected; therefore, the sensor should be placed as close as possible to the discharge source to ensure effective signal acquisition. Specifically, the #1 signal has a short wavefront and discharge duration and then returns to its initial state after an attenuated oscillation phase. In contrast, the #2 signal exhibits a slight oscillation process at the wavefront, with a very short wavefront time and discharge duration, after which the signal enters a long and steady oscillation phase following attenuation. In addition, the #2 signal has a short duration, which means that the #2 sensor did not detect the complete signal, which also proves that it is necessary to place two sensors. The point defect has an extremely small radius of curvature, causing significant electric field distortion, a small discharge area, and rapid energy release, resulting in a shorter duration of the PD signal. Additionally, due to the catadioptric effect of acoustic waves, a low-intensity signal is continuously detected at the tail of the #2 signal. This indicates that at greater distances from the PD source, the acoustic signals are more susceptible to fold-reflection effects, leading to a prolonged oscillation process.

The spectrum of the point discharge signals is analyzed, as shown in Figure 8b. The spectra of both the #1 and #2 signals are concentrated below 60 kHz, and both have a clear main peak near 10 kHz. Notably, the #1 signal has a main peak at 10 kHz and several secondary peaks near 20 kHz and 40 kHz, showing obvious frequency characteristics. Due to the higher attenuation coefficients of high-frequency signals in silicone rubber [42], the ultrasonic signals are predominantly concentrated in the low-frequency region. This observation is in good agreement with the results reported in [28], which investigated partial discharge signal detection in cable insulation systems.

### 4.3. PD Results of Air Gap Defect Tests

**Figure 9:**
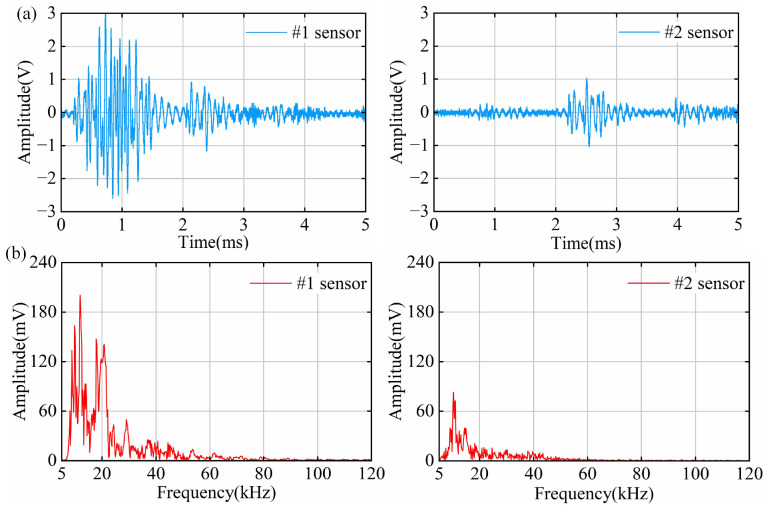
Time–frequency domain signals of PD in air gap defects: (**a**) time–domain signal diagram; (**b**) frequency–domain signal diagram.

The smallest average discharge amount detectable by the OFS within one minute is 26.0 pC. The time–domain waveforms of the air gap discharge signals measured by the OFS are shown in Figure 9a. The signal measured by the #1 sensor contains two consecutive discharge signals. The first has a long duration and a high discharge intensity; in contrast, the #2 sensor fails to detect the first and only detects the second one. Air gap defects have a weaker electric field distortion than point defects. PD is triggered when the voltage exceeds the insulation strength of the air gap. The electric field near the air gap defects is less concentrated and thus can store more energy. The discharge channel is formed after discharge, lowering the discharge threshold and enabling the discharge phenomenon with less energy.

The spectrum of the air gap discharge signal is analyzed, as shown in Figure 9b. The spectrum of the #1 signal is mainly concentrated below 50 kHz, with multiple main peaks distributed in the range of 10 kHz~20 kHz; since the first signal was not measured in #2, the spectrum is only distributed below 20 kHz, with the main peak located near 11 kHz. To further analyze the difference between the two discharges, spectrum analysis was performed separately, as shown in Figure S3. The spectrum of the first discharge is wider, and the second discharge is concentrated near 10 kHz, which can prove that the energy of the second discharge is weaker.

### 4.4. PD Results of Discharge Along Surface Defect Tests

**Figure 10:**
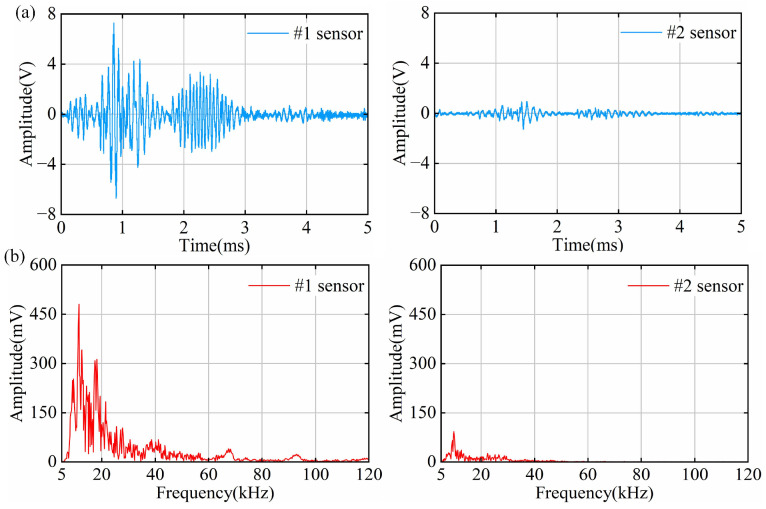
Time–frequency domain signals of PD along surface defects: (**a**) time–domain signal diagram; (**b**) frequency–domain signal diagram.

The smallest average discharge amount detectable by the OFS within one minute is 16.0 pC. The time–domain waveforms of the discharge along surface signals measured by the OFS are shown in Figure 10a. The #1 sensor captures two consecutive discharge signals. The first discharge has an obvious peak; the second discharge has no obvious peak, and both discharges exhibit a large energy release. In contrast, the #2 sensor detects only a weak signal. Discharge along the dielectric surface is often due to the elastic failure of the cable joints or a small amount of interference, resulting in the joints and cables not fitting tightly. At high field strengths, discharges along the direction of the joint surface are easily triggered. This study detected two discharge signals that had large energy and were continuous, indicating that the defects along the surface can store more charge. The weaker signal detected by the #2 sensor is because the ultrasonic signal generated by the discharge along the dielectric surface mainly propagates in the axial direction, and the signal attenuates more as the longitudinal propagation distance increases.

The frequency spectrum analysis of the discharge along the dielectric surface signals is shown in Figure 10b. The frequency spectrum of the #1 signal is mainly concentrated below 50 kHz and still has some components at 70 kHz and 90 kHz, with a clear single peak at 11 kHz and 18 kHz; the frequency spectrum of the #2 signal only has a weak frequency component at 0~30 kHz.

### 4.5. PD Results of Joint Misalignment Defect Tests

**Figure 11:**
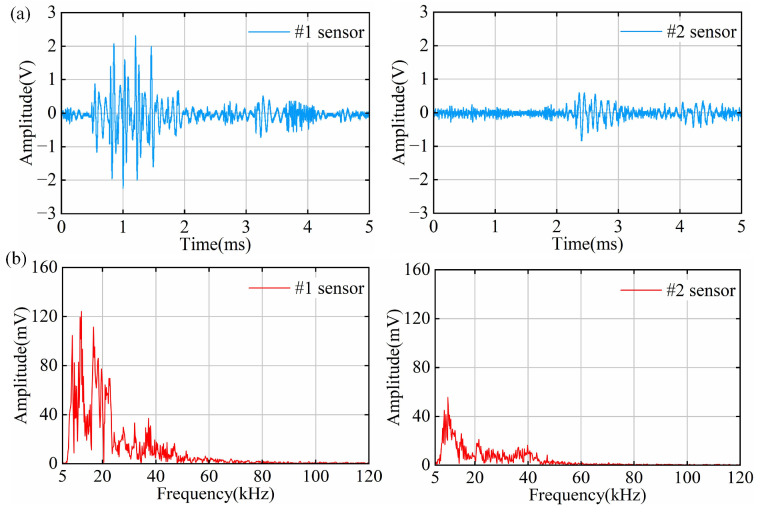
Time–frequency domain signals of PD in joint misalignment defects: (**a**) time–domain signal diagram; (**b**) frequency–domain signal diagram.

The smallest average discharge amount detectable by the OFS within one minute is 56.4 pC. The time–domain waveforms of the joint misalignment discharge signals measured by the OFS are shown in Figure 11a. The first discharge of the two sensors could not be well matched, indicating that the signals measured by the two sensors were not from the same discharge source. The #1 sensor recorded a series of complex discharge signals, whereas the #2 sensor captured a series of consecutive discharges with decreasing amplitude. As for the joint misalignment, the semi-conductive structure cannot effectively create a uniform electric field, and there is a concentration of the electric field both in the cable conductor and near the stress cone of the joint, leading to multiple discharge sources at distant locations. Therefore, the first signal measured by the #1 sensor contains multiple peaks that can be attributed to various sources of discharge within a small area of the conductor location.

The frequency spectrum analysis of the joint misalignment discharge signal is shown in Figure 11b. The frequency spectrum of the #1 signal is mainly distributed below 50 kHz, showing an obvious step; the #2 signal has a peak with a lower amplitude of 9 kHz. The frequency spectrum of multiple discharges of the #1 signal was analyzed separately, as shown in Figure S4. The first discharge was mainly concentrated below 25 kHz and showed multiple peaks; the subsequent discharge signals showed peaks at 37 kHz.

## 5. Conclusions

In this article, sensing optical fibers are implanted into 110 kV cable joints to construct cable joints that integrate both sensing and insulation performance. Relevant simulations are conducted, and a series of experiments is designed to verify the insulation and sensing performance of the optical fiber implantation method. The electric field distribution of optical fiber implantation between the insulating layer and the outer shielding layer of the cable joint is analyzed using finite element simulation. The results indicate that this implantation method has a negligible effect on the electric field distribution of the cable joint. Based on this implantation method, an optical fiber-implanted cable joint is fabricated and successfully passes the power frequency withstand voltage test and PD test. Subsequently, a platform for measuring the internal sound field response of the cable joint is established. Calibrated on a non-standard curved surface, the maximum average sensitivity of the OFS in the frequency range of 10 kHz~80 kHz is measured at 71.6 dB, which is 2.0 dB higher than that of a PZT. For 23 kHz ultrasonic signals, the peak frequency recorded by the OFS is 23 kHz, with an SNR of 65.2 dB. For 44 kHz ultrasonic signals, the peak frequency remains at 44 kHz, with an SNR of 64.9 dB.

Four typical discharge defects of cable joints are simulated: point discharge, air gap discharge, discharge along the surface, and joint misalignment discharge. Partial discharge detection experiments based on the optical fiber-implanted sensor are conducted. The optical fiber-implanted cable joint is capable of detecting a minimum average discharge amount of 16.0 pC within one minute. The results demonstrate that the optical fiber sensor exhibits high sensitivity and can effectively detect the characteristics of different types of partial discharges.

## Figures and Tables

**Table 1:** Dielectric parameters used for modeling the cable joint.

Material	Relative Permittivity	Conductivity (S/m)
semi-conducting silicone rubber	5.99	3.30
insulating silicone rubber	3.20	1 × 10^−18^
semi-conducting XLPE	2.30	2.30
insulating XLPE	2.60	1× 10^−18^
copper	1	6 × 10^7^
optical fiber	3.75	1× 10^−14^

## Data Availability

Data are available upon request from the authors.

## Supplementary Materials

The following supporting information can be downloaded at https://www.mdpi.com/article/10.3390/s25103166/s1, Figure S1: Fiber tail lead-out diagram; Figure S2: The results of the power frequency withstand voltage test and PD test; Figure S3: Frequency spectrum of a single discharge in an air gap measured by the #1 sensor; (a) frequency spectrum of the first discharge; (b) frequency spectrum of the second discharge; Figure S4: Frequency spectrum of a single discharge due to joint misalignment measured by the #1 sensor; (a) frequency spectrum of the first discharge; (b) frequency spectrum of the subsequent discharge.
