# Supplementary material for: Detecting Partial Discharge in Cable Joints Based on Implanting Optical Fiber Using MZ–Sagnac Interferometry
Source: Sensors (Basel). 2025 May 17;25(10):3166. doi: 10.3390/s25103166 (PMC12115856; doi:10.3390/s25103166)
Supplement: Supplementary file 1 [file sensors-25-03166-s001.zip › sensors-3647542-supplementary.pdf]

sensors

MDPI

Article

Detecting Partial Discharge in Cable Joints Based on Implanting Optical Fiber Using MZ–Sagnac Interferometry

Weikai Zhang [1], Yuxuan Song [1], Xiaowei Wu [2], Hong Liu [1], Haoyuan Tian [1], Zijie Tang [1], Shaopeng Xu [3] and Weigen Chen [1,*]

[1] State Key Laboratory of Power Transmission Equipment Technology, School of Electrical Engineering, Chongqing University, Chongqing 400044, China; wkzhang@stu.cqu.edu.cn (W.Z.); songyuxuan@cqu.edu.cn (Y.S.); liuhong@stu.cqu.edu.cn (H.L.); hytian@cqu.edu.cn (H.T.); tangzj0928@163.com (Z.T.)
[2] Shandong Taikai Cable Co., Ltd., Tai'an 271000, China; taikaiwxw@163.com
[3] School of Mathematics and Statistics, Hainan University, Haikou 570228, China; 994284@hainanu.edu.cn
* Correspondence: weigench@cqu.edu.cn

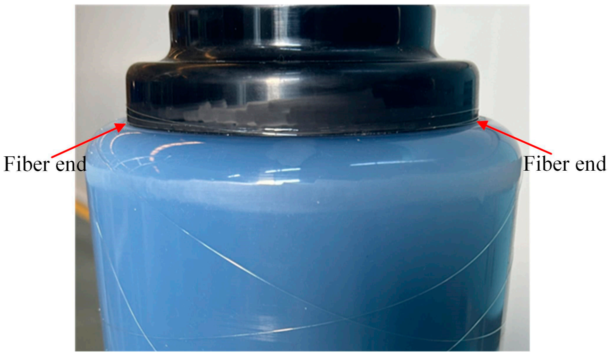

Figure S1. Fiber tail lead-out diagram.

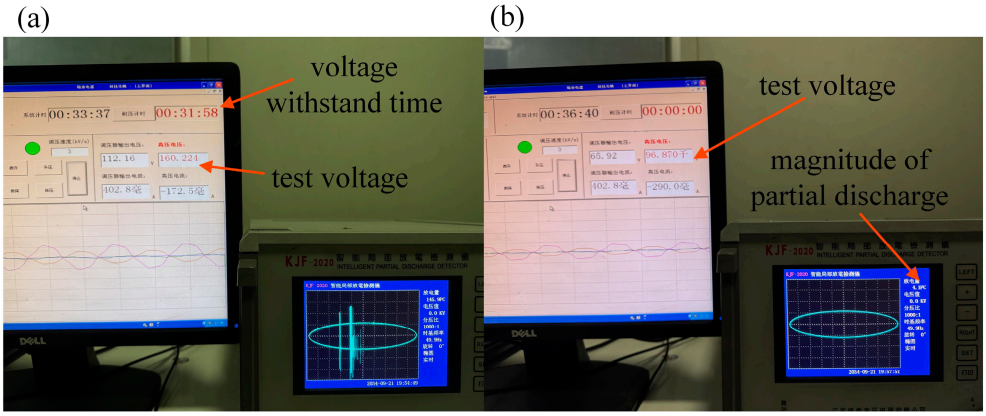

Figure S2. The results of the power frequency withstand voltage test and PD test.

 https://doi.org/10.3390/s25103166

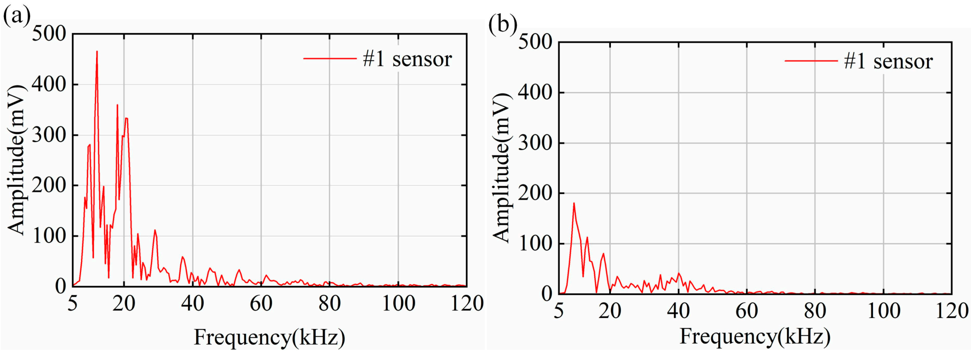

Figure S3. Frequency spectrum of single discharge in air gap measured by #1 sensor; (a) frequency spectrum of the first discharge;(b) frequency spectrum of the second discharge.

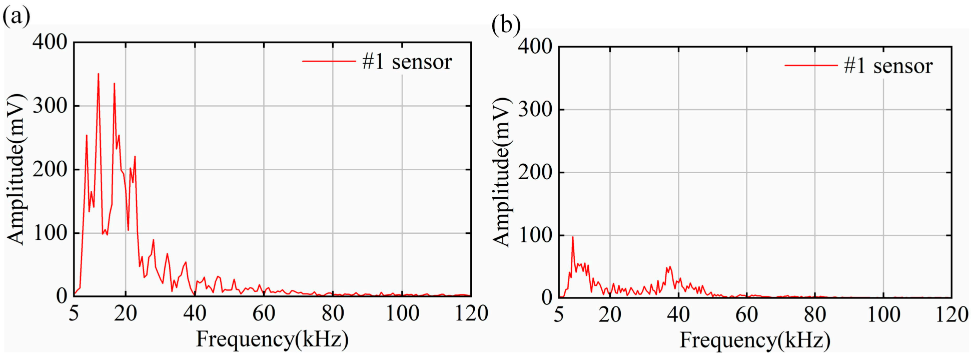

Figure S4. Frequency spectrum of single discharge due to joint misalignment measured by #1 sensor; (a) frequency spectrum of the first discharge;(b) frequency spectrum of subsequent discharge.

Author Contributions: Conceptualization, W.C.; methodology, W.Z., W.C., X.W., and Y.S.; software, W.Z., S.X., and Z.T.; validation, W.Z., H.L., and H.T.; resources, W.C., S.X., and X.W.; data curation, W.Z.; writing—original draft preparation, W.Z.; visualization, S.X., Z.T., and H.L.; writing—review and editing, Y.S.; project administration, H.T. All authors have read and agreed to the published version of the manuscript.

Funding: This research was funded by the Science & Technology Project of the State Grid Corporation of China, grant/award number 5108-202299263A-1-0-ZB. Shaopeng Xu was funded by the Hainan Provincial Natural Science Foundation of China (No. 122MS002).

Institutional Review Board Statement: Not applicable.

Informed Consent Statement: Not applicable.

Data Availability Statement: Data are available upon request from the authors.

Conflicts of Interest: Author Xiaowei Wu was employed by the company Shandong Taikai Cable Co., Ltd. The remaining authors declare that the research was conducted in the absence of any commercial or financial relationships that could be construed as a potential conflict of interest.
